# How funnel chanterelle (*Craterellus tubaeformis*) became an urban forager favorite in Scandinavia

**DOI:** 10.1186/s13002-025-00789-x

**Published:** 2025-05-27

**Authors:** Ingvar Svanberg, Mai Løvaas, Sabira Ståhlberg

**Affiliations:** 1 Institute for Russian and Eurasian Studies, Uppsala University, Box 514, 751 20 Uppsala, Sweden; 2 NTNU, Postboks 8905, N-7491 Trondheim, Norway

**Keywords:** Ethnomycology, Gastronomy, Leisure activities, Nordic cuisine, Urban foragers, Wild food

## Abstract

**Background:**

Peasants in preindustrial Norway and Sweden refused to touch edible macrofungi even during times of scarcity or famines, although this free food resource was abundantly available and authorities encouraged gathering mushrooms to enrich the diet since the eighteenth century. Urbanization and gradual changes of attitudes have turned mushroom gathering in forests and meadows into an important leisure activity. In recent decades, city foragers have discovered the funnel chanterelle, *Craterellus tubaeformis* (Fr.) Quél. This delicious edible mushroom has quickly become one of the most popular species for hobby gatherers. Our article follows the journey of the funnel chanterelle from an ignored food resource to a popular seasonal delicacy served also in luxury restaurants, discussing how, when and why attitudes and habits have changed.

**Methodology:**

For the historical background, this qualitative ethnomycological study uses a rich corpus of newspapers in the Swedish and Norwegian newspaper databases at the Swedish and Norwegian National Libraries. Data on contemporary mushroom hunter knowledge of *C. tubaeformis* have been obtained from responses to a questionnaire from 2017 with a hundred respondents. The study has also benefited from the authors’ participatory observations, own experiences as mushroom gatherers, and conversations with mushroom pickers in Norway and Sweden. Cookery books, mushroom identification guides and other printed works have also been utilized.

**Results:**

Urbanization caused a change in the relationship with nature: urban foragers are a fairly new phenomenon in Sweden and Norway but they have significant impact on food habits. City foragers discovered and have focused extensively on the funnel chanterelle ever since the end of the 1970s. It is now one of the most popular edible mushrooms in Sweden and Norway, widely publicized in newspapers, discussed in evening classes for novice mushroom gatherers, and presented in books and TV and internet food shows. Media and in recent decades also internet can be identified as the main information sources for urban gatherers. Attitudes have changed among others due to transformations in lifestyle, internationalization, and the fashion of consuming more local foods, as well as a strong need for leisure and perceiving nature as the best place for it, and gathering as a meaningful activity in nature.

**Conclusions:**

The funnel chanterelle is easy to identify, harvest and prepare. It is regarded as wild food with a wide range of uses, harvested for both personal consumption and commercial purposes, and now well-integrated in the Nordic cuisine. The urban population perceives mushrooms and various other wild foods as a normal part of the diet and modern food, in contrast to their peasant ancestors who thought fungi were animal food only. Contemporary human-fungi relations in Scandinavia have multiple meanings, not only as a food source but also as a recreational activity, maintaining emotional ties to the forests and nature among a highly urbanized population.

## Introduction

The relationship between humans and other living organisms is constantly changing: the human-macrofungi relationships in the Nordic countries is a manifest example of transformations in attitudes and activities, and how approaches in neighboring societies may vary. Mushroom gathering in Sweden and Norway is a modern activity; in Finland and Estonia, it is traditional. Using folk-life records and historical sources, the ethnologist Brita Egardt showed that the population in preindustrial Sweden harbored a deep distrust of mushrooms as food. This changed among the urban middle class and industrial working population only after the two World Wars in the twentieth century [1]. Later studies confirm these findings [2, 3]. Fungi were not part of the human diet among the rural populations in preindustrial Scandinavia until the 1880 s [2, 3]; thus, Scandinavia could be characterized as what social scientists label as traditionally mycophobic societies [4]. A consequence of these negative attitudes was that individual fungal species did not receive folk names, in contrast to vascular plants, for example [5]. Macrofungi mostly lack vernacular names in the Scandinavian languages. Possibly some boletus species were referred to as *kosvamp* “cow mushrooms” because peasants noted that cattle ate them, which gave an undesirable taste to milk [1]. The Swedish folklorist and mushroom connoisseur Bengt af Klintberg expressed it succinctly: “Autumn after autumn, chanterelles, orange milkcup, tooth fungi and penny bun sprouted from the ground and rotted away without people paying them much attention.” [5]. This situation changed with urbanization and due to extensive education efforts for several centuries to teach the public about the usefulness of wild mushrooms as a free resource available for everyone. These efforts met with sturdy resistance from rural inhabitants and were hardly efficient until urbanization processes had begun [6, 7].

By the late nineteenth century, more than a century after the first efforts were launched by the authorities, urban dwellers began to collect and consume a few mushroom species, especially golden chanterelle, *Cantharellus cibarius* Fr., porcini, *Boletus edulis* Bull.*,* and some other taxa [6, 7]. Of the many edible species available in the forests, only a few were known and recognized by these foraging beginners. By the first half of the twentieth century, the urban middle class had accepted mushrooms as food and was closely followed by working classes. A few became connoisseurs but most limited themselves to a couple of recognizable and conspicuous taxa [7]. With increased interest in mushrooms as food, the need to expand and improve the nomenclature of mushrooms appeared in Scandinavia. Mycologists and philologists collaborated to name the previously nameless fungi at the beginning of the twentieth century, often using German models [5].

With the majority of the population living in towns and cities and increasing affluence in society in the 1970 s, interest in additional mushroom species increased. Culinary and educational efforts by cookery and mushroom experts, who skillfully utilized mass media, evening classes, and other means to inform and teach about mushrooms, contributed strongly to creating a mushroom trend [6, 7]. Public interest in mushroom hunting gained momentum, and by 1980, gathering had developed into a popular recreational activity in Sweden. According to one qualified estimate, more than fifty percent of the adult population was collecting mushrooms by then [8].

Still, there were species which had gone unnoticed so far, such as funnel chanterelle *Craterellus tubaeformis* (Fr.) Quél. (Syn. *Cantharellus tubaeformis* and *Cantharellus infundibuliformis*) [9]. The fruit bodies grow abundantly in clusters and the taxon became very popular once it was discovered and enjoyed by urban foragers in the late 1970 s and early 1980 s. Today, it is one of the most common species in gatherers’ baskets, and it was also approved as an edible species by health authorities [2, 7]. In 2004, the Norwegian Food Safety Authority listed funnel chanterelle as one of eight safe mushrooms to identify, pick, and eat [10]. The species has a large content of bioactive compounds beneficial for human health [11].

Today, approximately forty percent of the current Swedish population aged 18–74 engage in fungi gathering. Most popular species are golden chanterelle*, Cantharellus cibarius* Fr., penny bun, *Boletus edulis* Bull., funnel chanterelle, *Craterellus tubaeformis* (Fr.) Quél., horn of plenty, *Craterellus cornucopioides* (L.) Pers. and yellow foot, *Craterellus lutescens* (Fr.) Fr. (Table 1) [7]. Specific data for the Norwegian population is not available, but gathering mushrooms is very popular also in Norway [12]. Research indicates that when food prices increase, the interest in mushroom collection also rises. The funnel chanterelle was one of the most commonly collected mushrooms in autumn 2024 [12].

**Table 1:** Contemporary names for most popular edible fungi in Scandinavia (Source [7])

Cantharellus cibarius Fr., Norwegian and Swedish* kantarell,* English* golden chanterelle*
Boletus edulis Bull., Norwegian *steinsopp*, Swedish karljohanssvamp, *stensopp*, English* penny bun*
Craterellus tubaeformis (Fr.) Quél. Swedish *trattkantarell*, Norwegian *traktkantarell*, English* funnel chanterelle*
Craterellus cornucopiodes (L.) Pers. Swedish: *svart trumpetsvamp*, Norwegian:* svart trompetsopp*, English *horn of plenty*
Craterellus lutescens (Fr.) Fr. Swedish *rödgul trumpetsvamp*, Norwegian *gul trompetsopp*, English *yellow foot*

In the following, we examine how funnel chanterelle developed from an ignored forest food resource to a popular ingredient, and its current significances for Scandinavians. This study falls within the field of ethnomycology, a rapidly growing area of research, which explores cultural aspects of human-fungi relationships [4, 13, 14].

### Biology and taxonomy

**Fig. 1:**
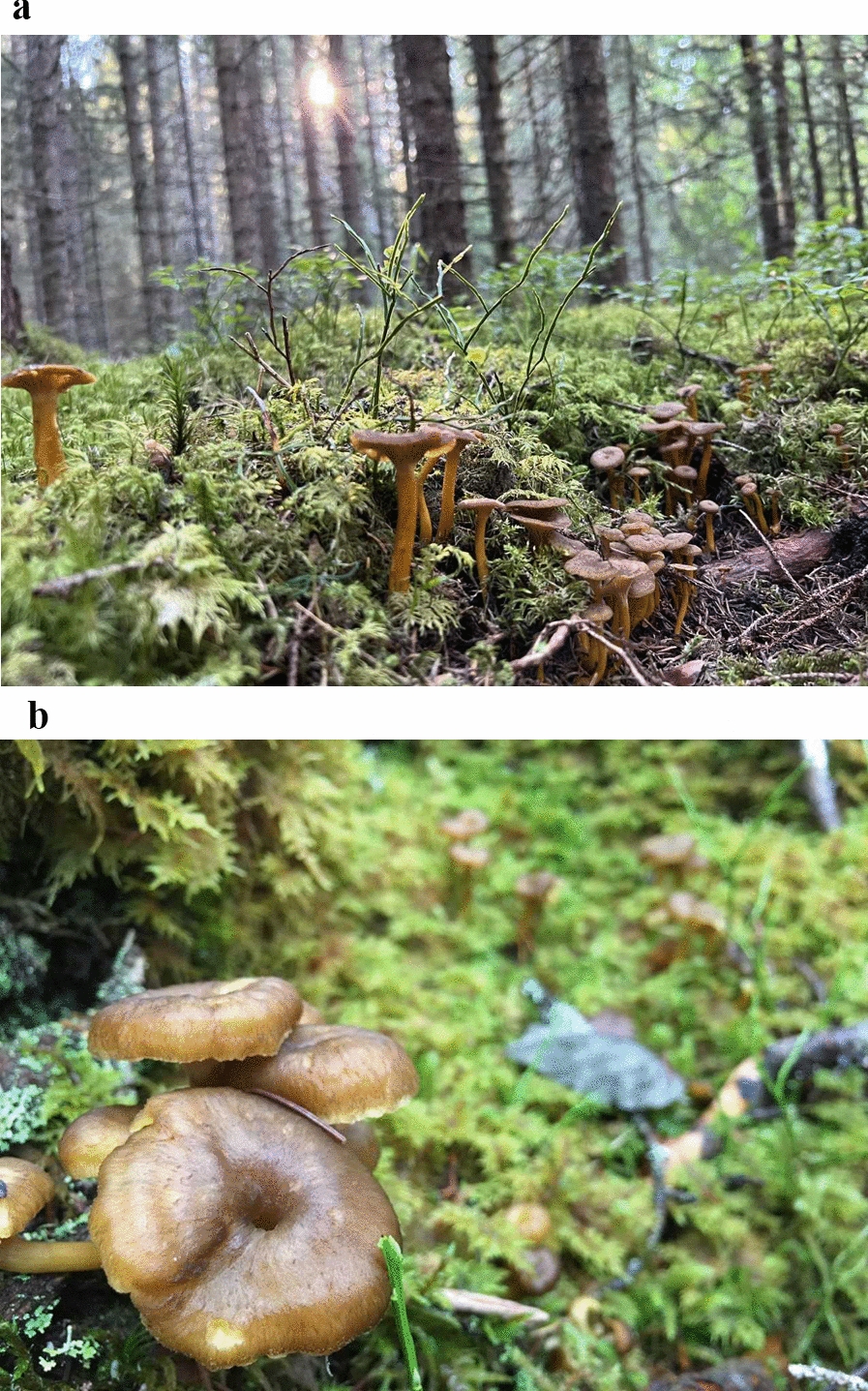
**a** and **b**. *Craterellus tubaeformis* in its natural habitat among mosses in a Norwegian forest. (Photograph: Mai Løvaas)

Funnel chanterelle, *Craterellus tubaeformis* (fam. Cantharellaceae), is an edible fungus [9], also known as trumpet chanterelle, winter chanterelle, and winter mushroom in English [15]. (Fig. 1a and b) Its common Swedish name is *trattkantarell*, first documented in 1896 in a Swedish booklet about mushrooms [16]. It has no traditional folk name and went long unnoticed by the peasantry. Like most names for edible macrofungi in Scandinavia, it is a neologism meaning literally “funnel chanterelle”, a word created by authors of mushroom field guides and handbooks [17]. Today it is often colloquially nicknamed *trattis* by gatherers. In Norwegian it is known as *traktkantarell,* first recorded in 1936 [18]. Despite this name, it is not an authentic chanterelle (of the genus *Cantharellus*) but belongs to a separate genus [19].

The funnel chanterelle is smaller than the golden chanterelle and harder to spot, as it has colors blending with the environment. Like golden chanterelles, it has chanterelle ridges and folds on the underside of the cap. Funnel chanterelles are thin-fleshed with a hollow, yellow stipe. The cap is deeply funnel-shaped, dark brown, 2–7 cm wide with light gray-yellow ridges. The flesh is insubstantial and brownish to yellowish. It can easily be confused with the yellow food, *Craterellus lutescens* (Fr.) Fr., also edible and popular among gatherers. The underside of funnel chanterelle contains ridges clearly demarcated toward the stipe, while a more indistinct distinction is formed in the yellow food, and the ribs are also flatter and more indistinct in the latter [20].

**Fig. 2:**
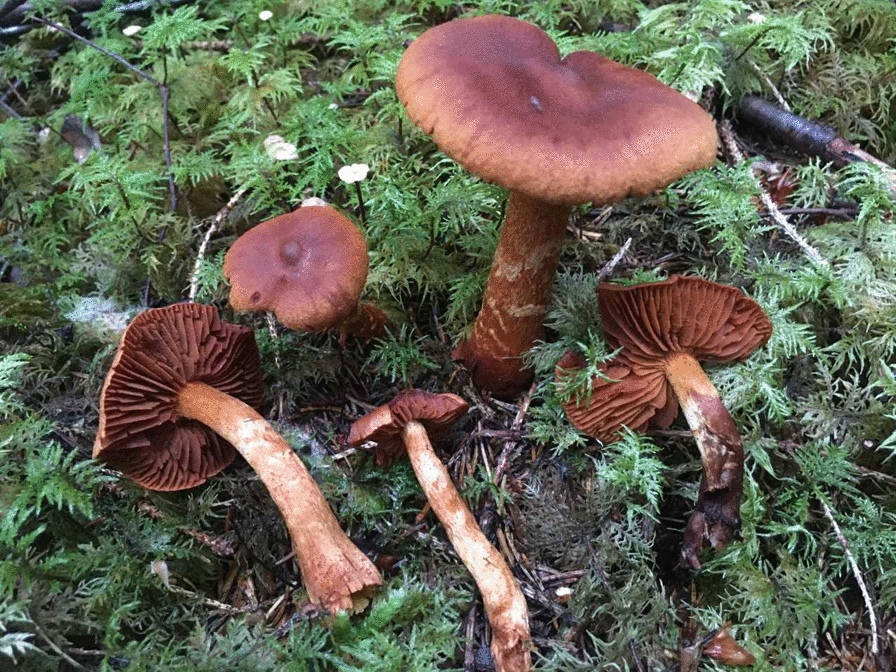
Deadly poisonous webcap*, Cortinarius rubellus*, can be mistaken for funnel chanterelle. (Photograph: Mai Løvaas)

**Fig. 3:**
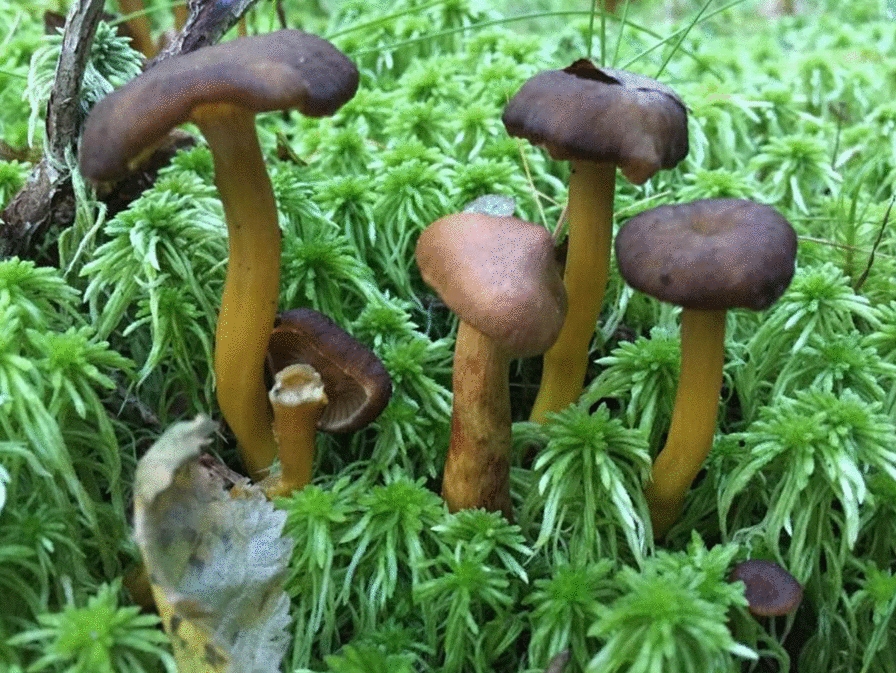
Norwegian mushroom gatherers learn quickly that they must pick funnel chanterelles one by one: as these grow so plentifully and close together in the forest, it is tempting to “rake” them up into the basket. But the extremely poisonous webcap, *Cortinarius rubellus*, can grow right in the middle of a cluster of funnel chanterelles, as seen here. (Photograph: Nikolai Kolstad)

Foragers sometimes confuse funnel chanterelle with the deadly poisonous webcap, *Cortinarius rubellus* (Cooke), despite different appearances [21]. Webcap grows in a similar habitat and sometimes even among funnel chanterelles (Figs. 2 and 3). Every year, there are consumers seeking treatment in Sweden for mushroom poisoning caused by mistaking webcap for funnel chanterelles [22]; cases are reported also in Norway. *C. rubellus* is known with the dysphemism “Sandefjord chanterelle” among mushroom gatherers in the south of Norway, because a person collecting mushrooms in the area of Sandefjord mistook *C. rubellus* for *C. tubaeformis* and allegedly had to get kidney dialysis for the rest of their life [23]. The Norwegian Association for Mycology and Foraging has a rigorous study program to certify mushroom experts who, on a volunteer basis, offer to check the baskets of mushroom foragers [24]. On multiple occasions in late autumn, extremely toxic *C. rubellus* have been found among funnel chanterelle, and mushroom experts must discard the entire contents for fear of contamination. *C. tubaeformis* can also be confused with the tasteless jelly baby, *Leotia lubrica* (Scop.) Pers*.* Its jelly-like texture, however, is a clear differentiator.

**Fig. 4:**
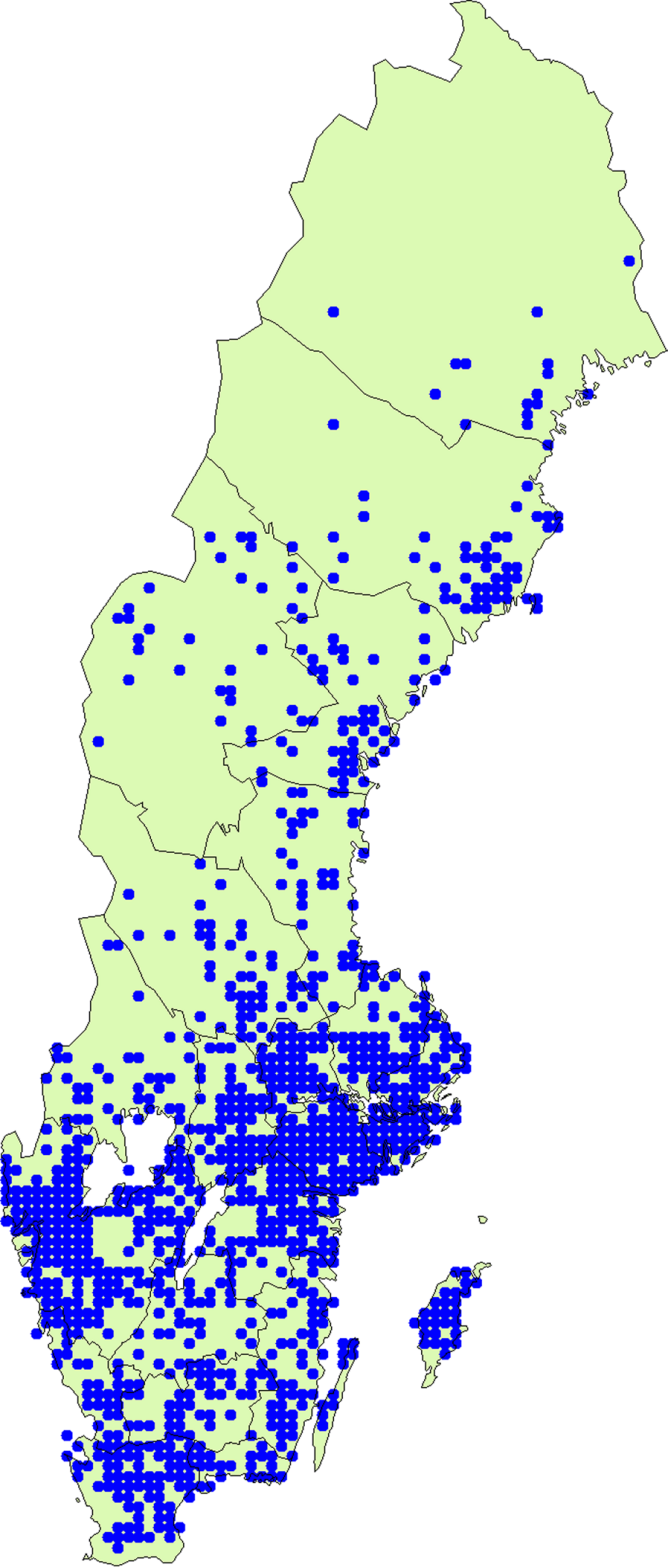
Reported observations of *Craterellus tubaeformis* in Sweden (Source: SLU Swedish Species Information Centre)

**Fig. 5:**
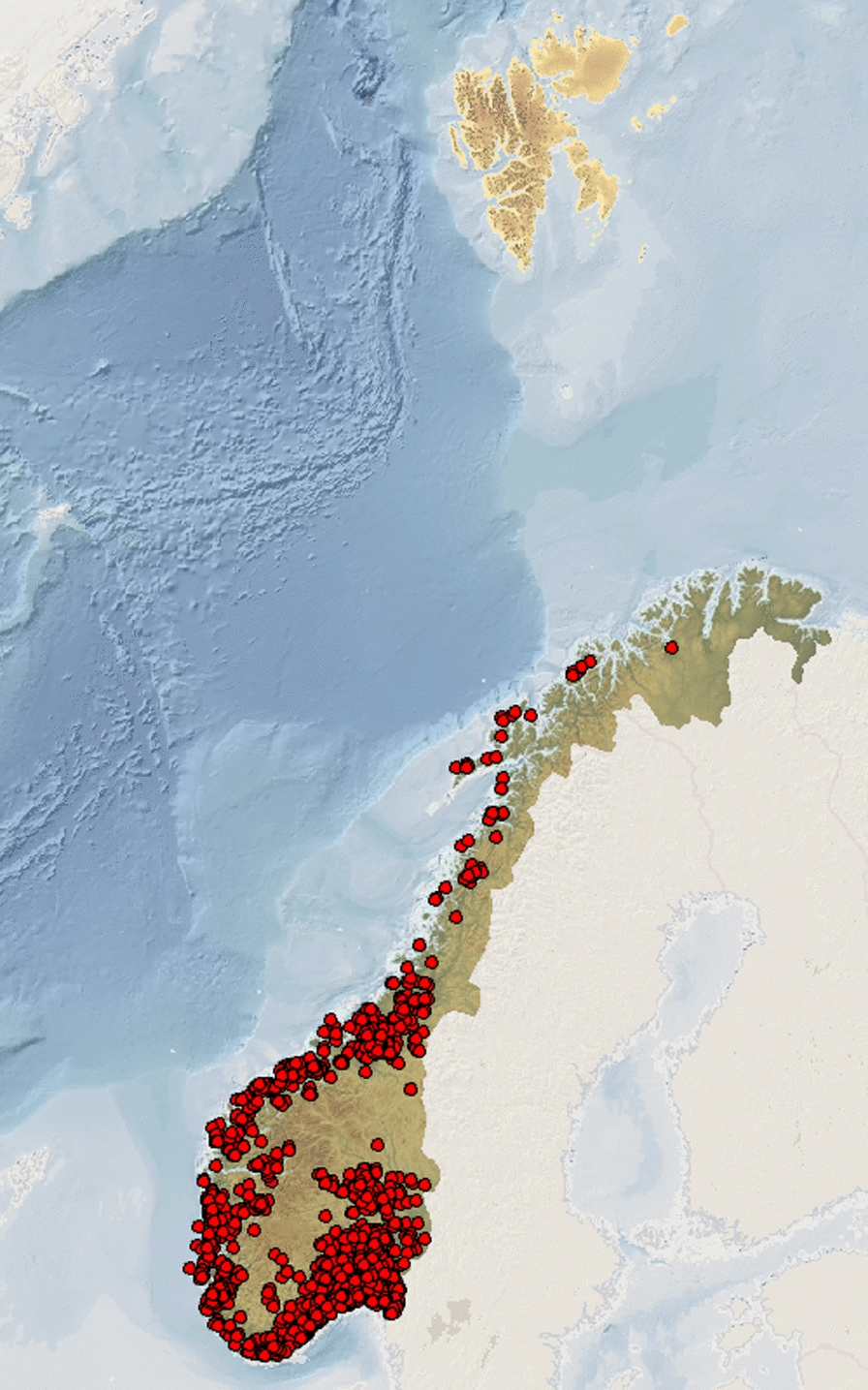
Reported observations of *Craterellus tubaeformis* in Norway (Source: Norwegian Species Information Centre)

Funnel chanterelle grows in moss in coniferous and beech forests. It is rarely seen in other deciduous forests, as it prefers acidic and lean soil. It grows in acidic, alkali, and nutrient-poor beech, pine, and spruce forests, on moderately to significantly moist soil, but it only appears on basic or neutral rock if acidic soil is present. Funnel chanterelle forms ectomycorrhiza with various conifers, especially *Picea abies* (L.) H. Karst, and *Pinus sylvestris* L., and occasionally also with deciduous trees. It forms fruiting bodies from August to November and sometimes even later, and in rainy weather, fruiting bodies can be found as early as July. Funnel chanterelle can grow in very large batches [25]. *Craterellus tubaeformis* is a widespread basidiomycete with a Holarctic distribution and is accordingly found in Europe, northern Asia, and North America. The funnel chanterelle is quite common in southern and central Sweden but is rarer in the northern part of the country [23]. In Norway, it is found in forest areas throughout the country (Figs. 4 and 5) [26].

## Methods and sources

This study focuses on human-mushroom interactions, gatherer behavior and knowledge, and perceptions of fungi from the eighteenth century until today. Qualitative ethnomycological research can offer extensive insights into the historical and contemporary relationships between humans and fungi [7, 13, 27, 28]. The complex relationship and transformations in attitudes necessitate the use of various methods in ethnomycological studies. We have analyzed through keywords the rich corpus of almost 1,600 newspaper titles from the seventeenth century until today in the database of the Swedish Royal Library (tidningar.kb.se). Norwegian newspapers were searched at the database provided by The National Library of Norway (www.nb.no/search?mediatype=aviser). Especially in autumn, articles, reports and recipes concerning mushrooms are abundantly published in Scandinavian newspapers. Through a diachronic perspective, we can outline at what time in history *C. tubaeformis* became accepted as an edible mushroom. Further, our approach relies on a rich empirical narrative [29]. For the present-day discussion, our data concerning Swedish contemporary mushroom gathering, and *C. tubaeformis* specifically, originate in our questionnaire prepared for the autumn mushroom season 2017. The context, selection of participants, and their answers were reported in a comprehensive overall study of mushroom picking in Sweden in 2019 [7]; here, it is important only to mention that of 100 respondents, 56 were women and 44 men. Most lived in larger cities but had grown up in smaller communities or in rural areas; a majority of respondents were highly educated. In addition to the usual background information (age, place of residence, gender, urban/rural background), the questionnaire included questions about how often the respondent eats wild mushrooms; species; favorites; which mushrooms were collected in their youth; how they learned to recognize wild mushrooms; the importance of wild mushrooms in their household; which mushrooms they collect; ways of preparation and preservation methods; participation in courses, and contact with mushroom consultants. The questionnaire answers provide information concerning when, where and by whom C. *tubaeformis* was collected, and for what purposes the mushroom harvests were used. Cookery books, mushroom identification field guides, scientific reports, and historical studies have also been useful for our knowledge [6, 7], as were online databases like AnthroSource, Sciencedirect and SCOPUS, using keywords such as foraging, fungus, mushroom, non-timber products, and wild foods for comparative studies.

To understand how mushroom gatherers perceive species in various habitats, this study has benefited much from our participant observations, conversations with foragers in Norway and Sweden, and exchanges with hundreds of gatherers in Sweden, Norway, Finland, and internationally, over several decades and in various situations. As commonly with observatory participation, gathering of information and exchanges over such long periods, it is impossible to provide concrete figures of participants, protocols, and exact scientific data. Yet, in ethnobiology it is important to acknowledge that *experience-based understanding and knowledge* are highly significant. Many of the persons we have talked with over the years, often in sudden and unexpected encounters on a forest path or in a field while looking for mushrooms, have shared their experience. They would never participate in regular field work inquiries or reply to a questionnaire, but their knowledge has been crucial for our understanding about mushrooms. Also, the benefit of being a participant for a long period of time is that the knowledge we constantly collect is processed in various ways, and therefore can become both broader and deeper than time- and space-limited scholarly fieldwork.

## Results

Norway and Sweden share a border of 1,630 km, similar political systems, mutually intelligible languages, and minor cultural differences. Sweden has 10.6 million inhabitants, nearly double in comparison with Norway’s 5.6 million (2024). In Scandinavia, the so-called “Everyman’s Right” (Swedish *allemansrätten*, Norwegian *allemansretten*) permits public access to the wilderness for outdoor activities and gathering mushrooms, wildflowers and wild berries. This is an important traditional and statutory right, fundamental in society and taken for granted by the citizens [30, 31].

Outdoor activities and recreation play a significant role in the culture of Scandinavian countries and for the well-being of the people. Despite being highly urbanized, they maintain a deep emotional relationship with forests. Fishing and hunting are popular but even more common is foraging. In contrast to fishing and hunting which require paid permits, gathering does not put any strain on the household budget. Most people collect berries and mushrooms for recreation [2, 3, 7, 12, 31]. Many of our informants emphasize in the questionnaire that “just by being in the forest” they feel it is good for their health and relaxation; we can confirm this from our experiences. Mushrooms or berries are a bonus; people are not disappointed even if their harvest is poor.

While berry-picking has a history going back to early rural society [2, 8], the interest in foraging edible mushrooms is clearly a result of urbanization and modern education efforts [7]. In Norway in 1902, the society *Landsforeningen til Udnyttelse af vore Nyttevexter* (The National Association for the Utilization of our Useful Plants) was established; today it is called The Norwegian Association for Mycology and Foraging. In 1936, the society published a mushroom name list, containing 400 Norwegian names created and invented by mycologists. This is the first documentation of *C. tubaeformis* in Norway [32]. The same year it was included in Chr. Fr. Bøhme’s handbook under the scientific name *Cantharellus tubaeformis* and named *trompetkantarell “*trumpet chanterelle” [33]. His idea was to turn public interest toward foraging edible species in Norway, as there was an abundance of food in the forests; thus, in his view, the country had no need to import wild plants and mushrooms from abroad.

Fungi handbooks have been crucial for spreading the knowledge about edible species. In Sweden, a mushroom enthusiast, Reverend Nils Gustaf Strömbom (1847–1897) was the first to mention funnel chanterelle in a handbook published in 1896 [16]. He says nothing about its use as human food, however. With growing urbanization from the end of the nineteenth century, not only the distance between humans and the forests increased, but also newspapers became more influential, reaching far more people than before. In the urban context, daily newspapers and weekly magazines are still important in disseminating edible mushroom information and recipes. Our review of newspapers shows that well-known Swedish mushroom enthusiasts like Bengt Cortin (1907–1960) wrote about the funnel chanterelle as early as 1936 [34]. Another well-known mycologist, Nils Suber (1890–1985), wrote about its use as food in 1958 [35].

Searching with keywords and key concepts through newspaper databases may naturally cause slips, as no search engine is perfect, but considering the published mentions of funnel chanterelle above, and the fact that there is no earlier folk name for the mushroom, we can fairly safely assert that the mushroom is absent from older newspapers. Analysis indicates that there were occasional articles attempting to spread knowledge of funnel chanterelles in the 1960 s in Sweden, but it was only in the 1970 s that these edible fungi were specifically promoted as healthy and delicious food. From 1975 onward, funnel chanterelle was mentioned as an excellent edible mushroom in daily newspapers each autumn. In 1979, there were eleven articles, but in the following years up to a hundred articles mentioning funnel chanterelles were published annually. In 1984, a Swedish mushroom book for children was published with funnel chanterelle in the title, indicating, if nothing else, its full acceptance as a popular food fungus [36]. The rise has not been even and there are waves of interest: during the 2010 s, there was a marked increase from 43 hits in 2010 to 376 articles in 2013, and the following year to 830. After that, the number of articles has remained at a constant high level (690 in 2023). Nowadays, the interest in this taxon and other species is promoted in various kinds of social media as well [7].

An extensive search of newspapers in The National Library of Norway shows that the first mention of funnel chanterelle was in 1943 [37]. A wartime article directed at gatherers contained a list of prices for commercial mushrooms; fungi were seen as good subsistence food during this crisis with compulsory food rationing. Funnel chanterelle was ranked in category II, where a gatherer received 3 NOK per kilogram for selling to factories; NOK 3,50 for selling to a distributor; or if sold directly to consumers on the market, NOK 4,75 per kg (conversion rate in 1945: 1 NOK = 0,21 USD). The next time funnel chanterelle was mentioned was in 1945, a year of drought and few mushrooms, although there were hopes for a late season with rains and subsequently funnel chanterelles [38]. The article states that in a famine during the war people collected mushrooms for food. Since the war was now over, the authors assumed that there would be fewer mushroom gatherers, but that there would still be people aware of mushrooms as a good source of free and enjoyable food [38]. From 1951 to 1977, funnel chanterelle was mentioned between one and seven times per year, and in 1978 it was discussed 19 times. Until 1986, articles appeared between four and 17 times per year, and in 1987 it was mentioned 56 times. From the year 2000 until 2022, there were between fifty and a hundred articles in newspapers, with a peak in 2011 with 104 articles and a newspaper reporting that “we are standing in a sea of funnel chanterelles” [39].

### Acceptance as food

**Fig. 6:**
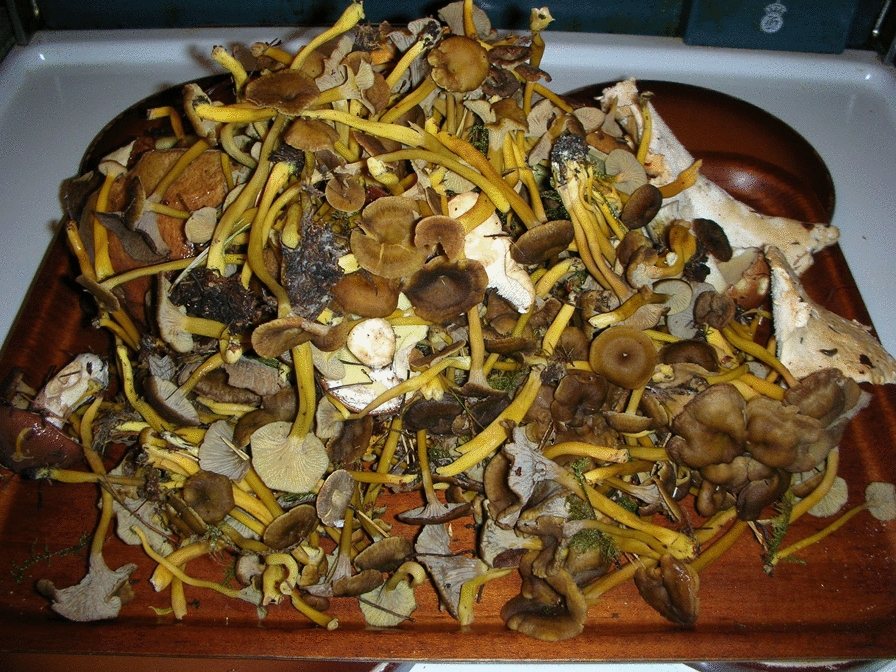
Newly harvested funnel chanterelle ready to be cleaned (Photograph: Maj Reinhammar)

Funnel chanterelle clearly gained popularity as food in the early 1980 s, as seen above, but the interest among recreational foragers in *C. tubaeformis* in Sweden decreased radically after the 1986 Chornobyl’ (Chernobyl) disaster in Ukraine, due to high levels of Cesium-137 found in mushrooms [40, 41]. Although the Swedish Radiation Safety Authority still carry out measurement projects to map the levels of cesium-137 in mushrooms [41], our informants no longer worry about it. Within a few years the species regained its popularity, and in our questionnaire it came third in popularity after *Cantharellus cibarius* Fr., and *Boletus edulis* Bull. [7]. In a study from the Swedish province of Småland, published in 2015 and based on 60 interviews, it was ranked second among popular food fungi [42]. Annual observations among approximately twenty vendors selling fungi at a market in Uppsala, Sweden, confirm that between 2008 and 2024, funnel chanterelles are among the most commonly marketed edible mushrooms, in stiff competition with the golden chanterelle, porcini, and black trumpet, *Craterellus cornucopioides* (L.) Pers. Funnel chanterelle is not only picked for domestic consumption but also commercially: it is commonly available in autumn at street markets and in grocery stores. Seasonal workers, mainly from Southeast Asia, arrive in Sweden as pickers for the mushroom industry [2, 7]. *C. tubaeformis* is free wild food, of high culinary quality, and versatile, as it can be prepared in many ways, and it can also be frozen or dried. (Fig. 6)

**Fig. 7:**
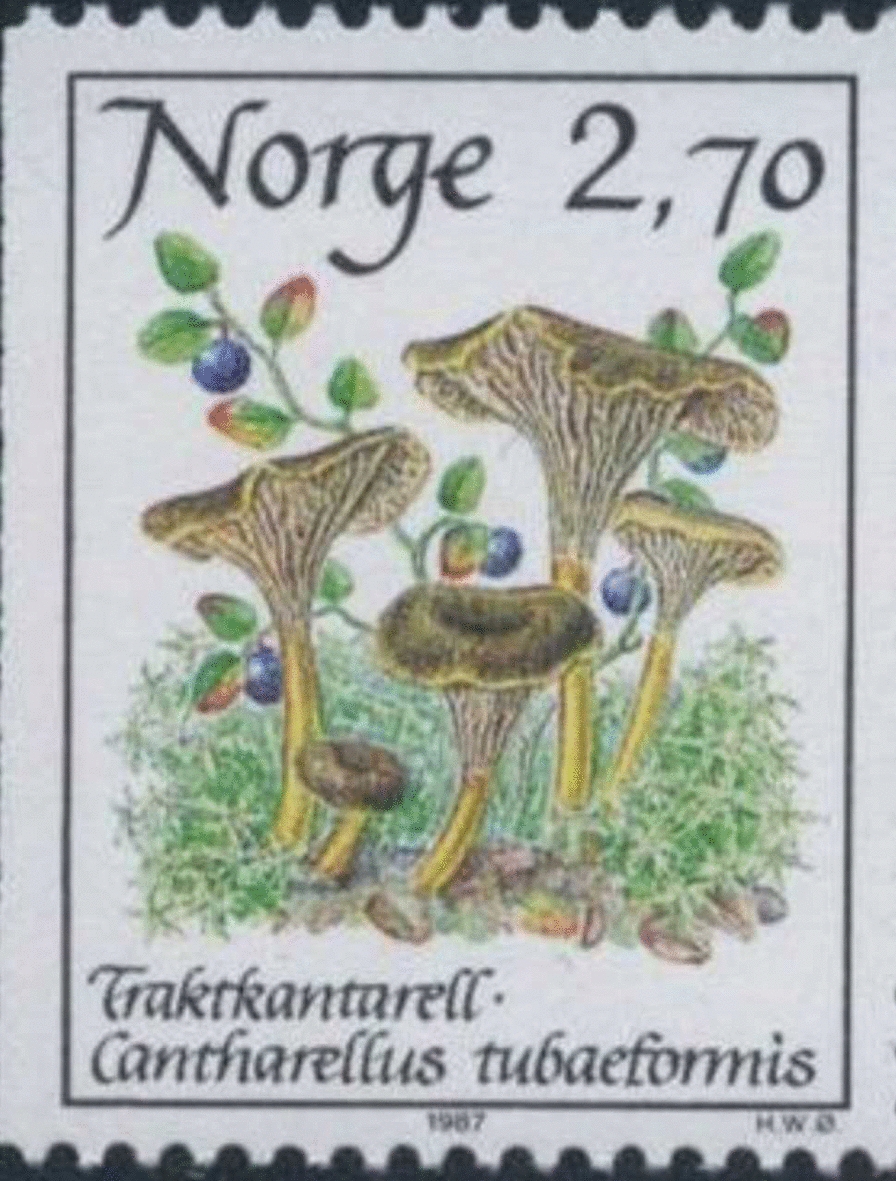
Norwegian stamp depicting funnel chanterelles issued in 1987

To increase the awareness of edible fungi, mushrooms as symbols for provinces were designated in Sweden in the 1980 s in consultation with mushroom activists. The funnel chanterelle is the “provincial fungus” of Västmanland in central Sweden [43]. In Norway, it was depicted on a stamp illustrating edible mushrooms issued in 1986 (Fig. 7). Nowadays, *C. tubaeformis* is one of the many edible fungi species harvested in Scandinavia [7, 44].

**Fig. 8:**
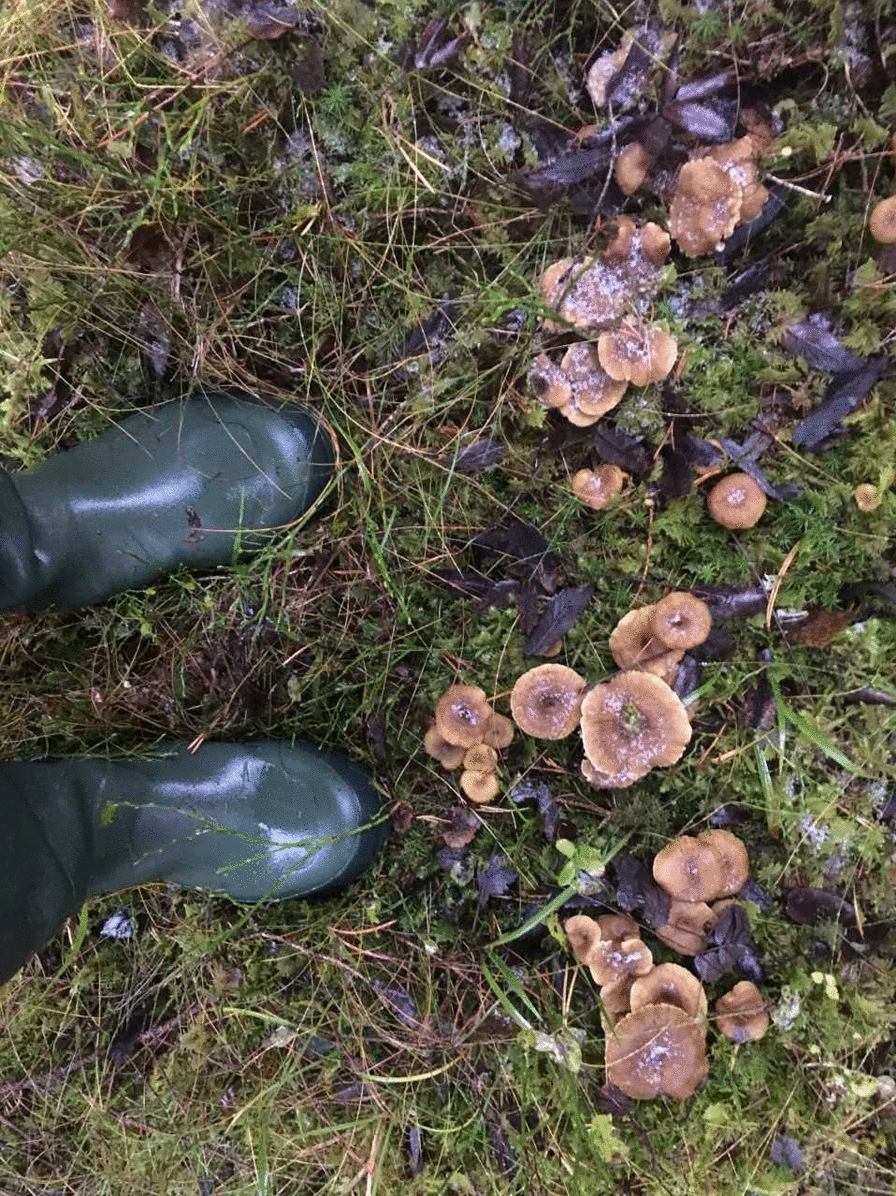
It may take some time for the mushroom gatherer to get used to seeing funnel chanterelles among the moss. There are usually plenty of them. These are frozen solid, but are not harmed and are still good to eat. (Photograph: Mai Løvaas)

Although *C. tubaeformis* is an important food source for other mammal species (elk, wild boar, roe deer), human mushroomers do not really compete with them. Only a small fraction of the *C. tubaeformis* growing in the forests is gathered; there is significant potential to increase the harvest. Although it is claimed that wild mushrooms contain beneficial minerals and nutrients, the strong, fine flavor of this species attracts the Scandinavian consumer and makes the funnel chanterelle highly popular; less than the fact that it is rich in vitamin D 2 (15.4 µg/100 gr.) [11]. A contributing factor besides availability and taste is that *Craterellus tubaeformis* grows and is harvested in late autumn, at the wane of the standard mushroom season, and it has a long and late season into early winter. Even at Christmas, people put on winter clothes and a headlamp and gather funnel chanterelle among patches of snow [21]. It has become a popular pastime for many foragers to harvest them over several weeks or months (Fig. 8).

### Trade and availability for consumers

**Fig. 9:**
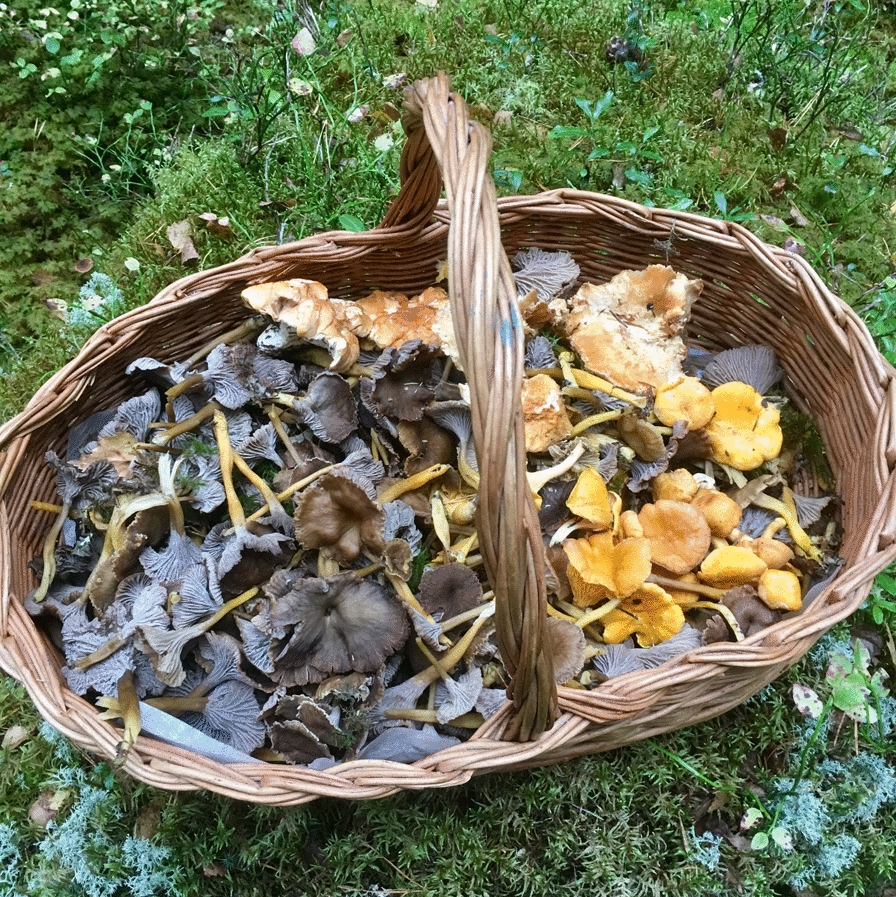
A traditional mushroom picker’s basket with a harvest of funnel and golden chanterelles in Storfors, Sweden, October 2017. (Photograph: Simon Sorgenfrei)

In addition to a few species of wild berries, golden chanterelle and funnel chanterelle are among the most important non-wood forest products harvested in Scandinavia also for commercial purposes [2]. Nevertheless, fungi remain an underutilized non-timber forest product. (Fig. 9).

Wild berries have increased in importance among consumers during the twentieth century due to the prevalence of cheap sugar [2]. Especially cowberry, *Vaccinium vitis-idaea* L., bilberry, *Vaccinium myrtillus* L., and cloudberry, *Rubus chamaemorus* L., are consumed on a large scale in contemporary Scandinavia and are also widely used in the food industry [2, 45]. Wild mushrooms have not reached food chain to the same extent. Grocery stores in Scandinavia mainly sell fresh cultivated mushrooms, but in autumn they also offer wild-picked golden chanterelles and funnel chanterelles from foragers. In contrast to many other European countries, there are no legal regulations restricting the trade with wild mushrooms in Sweden and Norway [12, 46]. Until December, fresh funnel chanterelles are often available in the shops [47].

**Fig. 10:**
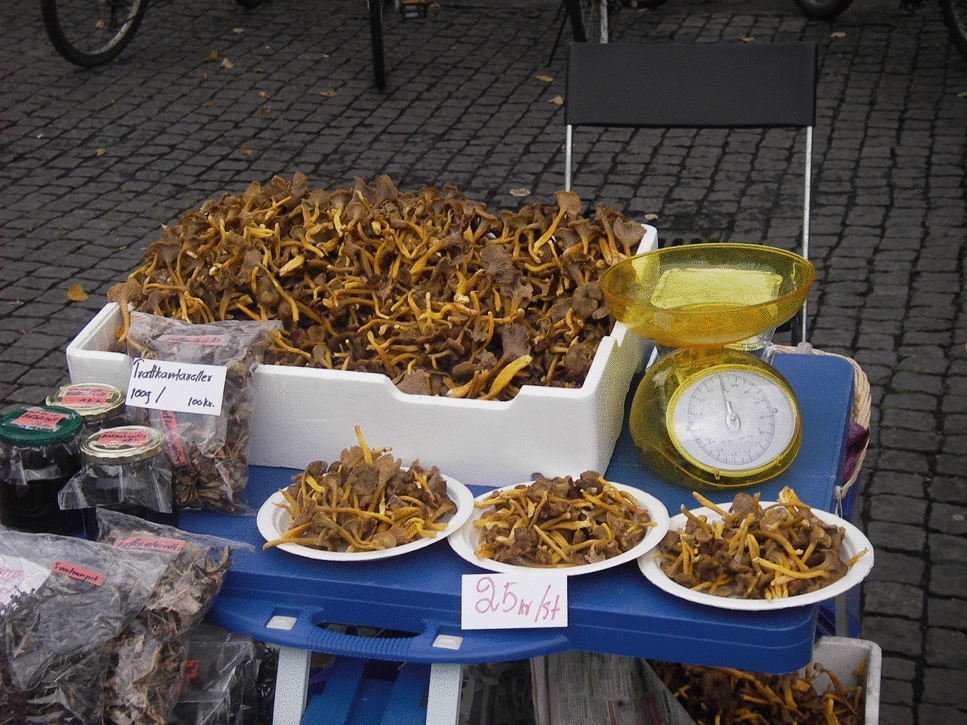
Funnel chanterelle sold at a Thai market stand in the weekly market at Vaksala torg, Uppsala, Sweden. (Photograph: Navarana Ingvarsdóttir Olsen, 2012)

Dried funnel chanterelles are available all year in well-equipped grocery stores. Funnel chanterelle is on List 1 that is a mushroom suitable for commercial purposes, in a 2012 report funded by The Nordic Council of Ministers [48]. In addition to grocery stores, from autumn until Christmas it is often available both fresh and dried at weekly markets where small-scale farmers and immigrants (mostly women) sell their produce. Especially Thai communities harvest and sell large quantities of funnel chanterelles and other species at popular weekly markets in many Swedish cities [2]. (Fig. 10).

**Fig. 11:**
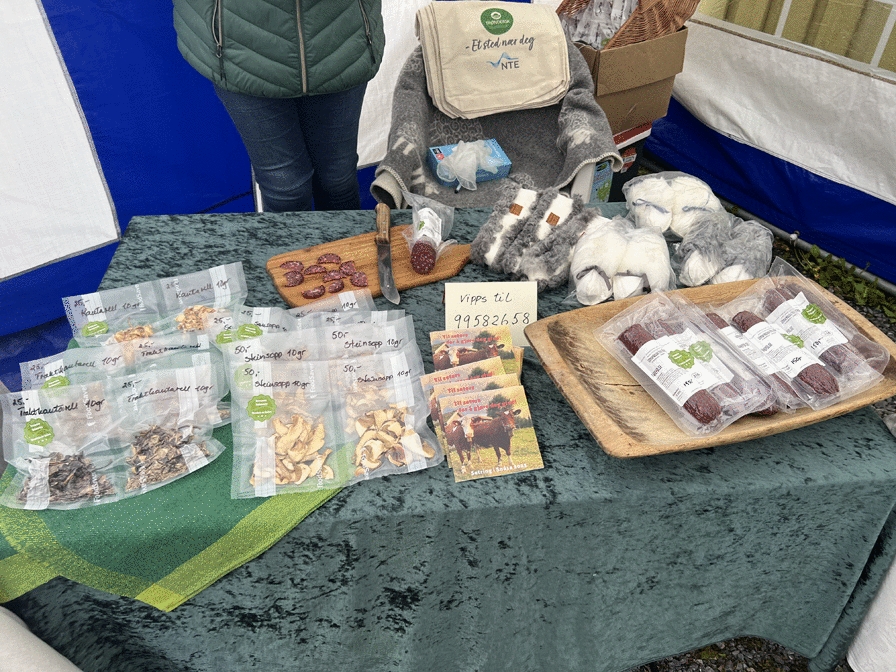
Dried funnel chanterelles sold in June 2023 at a food festival in Snåsa in Trøndelag, Norway, with the label “Smaken av Snåsa” (“Taste of Snåsa”). The seller gathers the mushrooms and puts them on display alongside other products. (Photograph: Mai Løvaas, 2023)

**Fig. 12:**
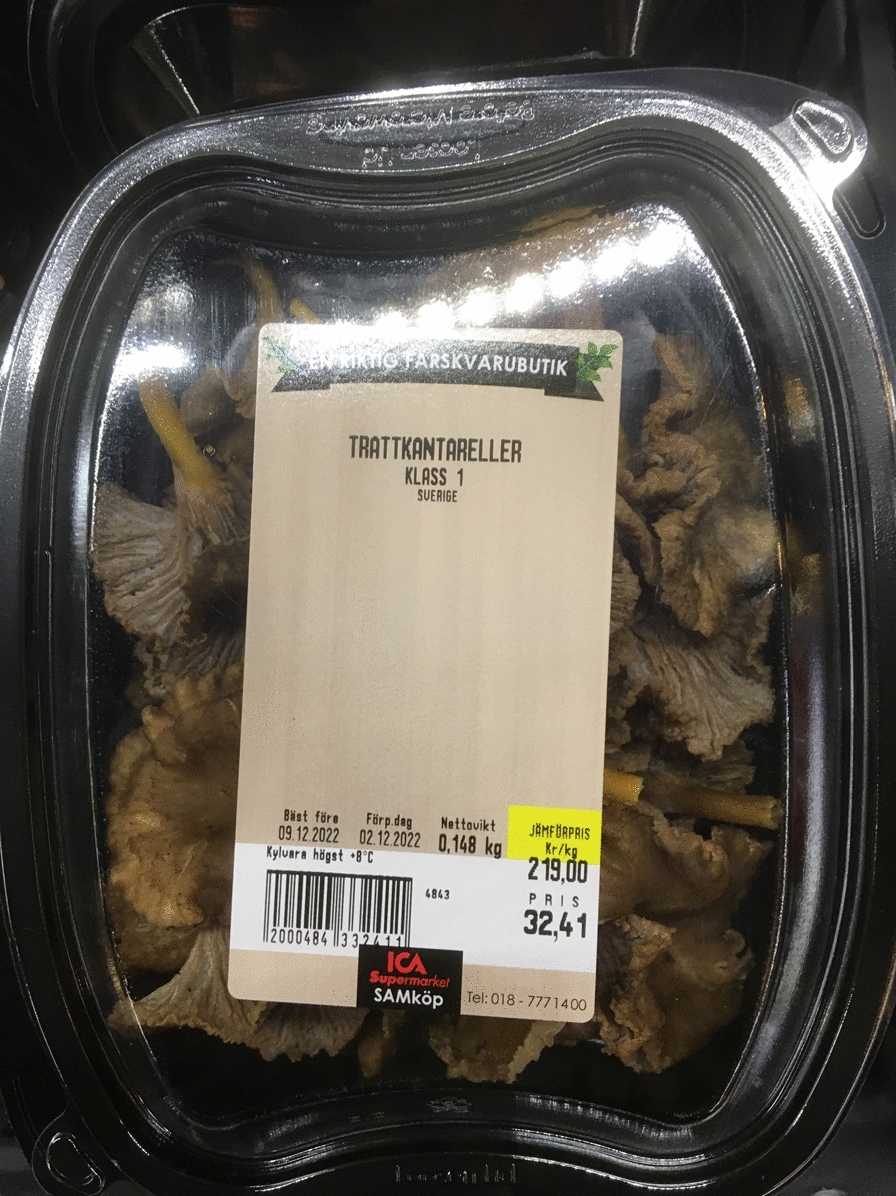
Funnel chanterelles sold in a grocery store in Uppsala, Sweden, December 2022. (Photograph: Ingvar Svanberg)

The funnel chanterelle is also sold dried in small paper bags in shops providing locally produced food. For consumers who do not gather mushrooms themselves, funnel chanterelles are easily available and often their gatherer friends bring the mushroom as gifts. In recent years, the range of cultivated mushrooms has increased significantly. A number of cultivated species are available in common grocery stores: button mushrooms, *Agaricus bisporus* (J.E. Lange) Imbach, oyster mushrooms, *Pleurotus ostreatus* (Jacq.) P. Kumm., and shiitake mushrooms, *Lentinula edodes* (Berk.) Pegler; all are grown in Sweden. Less common are wood-ear mushrooms, *Auricularia polytricha* (Mont.) Sacc. and enoki, *Flammulina filiformis* Z.W. Ge, X.B. Liu & Zhu L. Yang) P.M. Wang, Y.C. Dai, E. Horak & Zhu L.Yang, which are mostly imported. Button mushrooms are enjoyed by many consumers, used in modern home cooking, while many of the other species are used in Asian cooking and by immigrants. Mushrooms harvested from the wild are chiefly the golden chanterelle and funnel chanterelle. No official statistics on either commercial or private mushroom picking are available, however [12, 47]. (Figs. 11 and 12).

### Funnel chanterelle in contemporary Scandinavian cuisine

*Craterellus tubaeformis* can be used fresh or dried, and it is notably versatile in its uses; it can be stored frozen and maintains its rich flavor and quality also when dried. Dried mushrooms are soaked for thirty minutes in water, wine or milk before use, and when cooked, they retain their savory culinary qualities. A bag of dried funnel chanterelles is a popular giveaway in Scandinavia. Funnel chanterelle is described in current Norwegian cookbooks as “steadfast and reliable” and a mushroom which all foragers collect at least once a year [49].

**Fig. 13:**
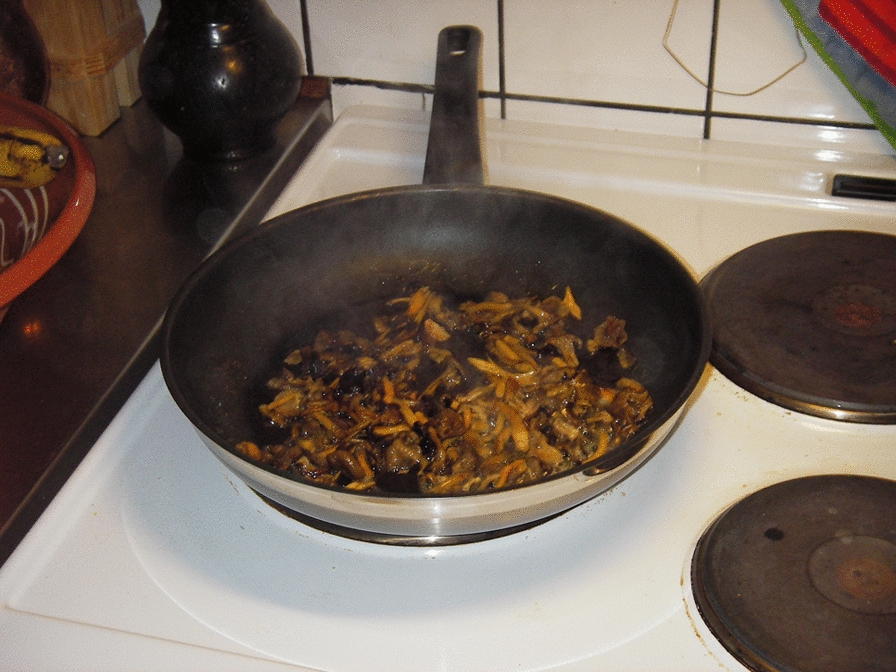
Funnel chanterelles are heated in a pan; this draws out the liquid from them; then, the mushrooms are stored or frozen in their own liquid to be used later. (Photograph: Ingvar Svanberg)

There are many recipes for funnel chanterelle in cookbooks, weekly magazines, newspapers, and on websites: funnel chanterelle sandwich is toasted bread with butter-fried mushrooms topped with fresh parsley [50]; funnel chanterelle caramel, funnel chanterelle ground and used as mushroom meal [51], and salmon fricassee with funnel chanterelle [49] are popular foods not only at home but also in lunch and fine-dining restaurants. It is used in household cooking for various dishes, soups, and sauces. It is often served with reindeer, elk, boar, and other game meats. The indigenous Sámi groups in northern Sweden and Norway have recently integrated it into their cuisine [52]. Reindeer stew with funnel chanterelle, and blue cheese with funnel chanterelle marmalade are just two examples [47]. The potential and possibilities for making a variety of dishes with this mushroom are extensive. (Fig. 13).

## Discussion

Ethnobiologists often discuss in regretting tones the loss of local or traditional knowledge when it comes to plant know-how among various peoples [53]. The case of funnel chanterelle in Sweden and Norway shows that knowledge changes with society, economy, lifestyle, globalization, industrialization, and food preferences. Even if certain sectors of knowledge are lost, as the living environment and food acquisition transform, other sectors are introduced and developed. Two hundred years ago, Scandinavian peasants utilized a wide range of plants and animals from their environment, but after urbanization gained speed more than a hundred years ago, various kinds of new knowledge, induced both internationally, nationally and locally, about food, nature, and uses of previously ignored species have appeared. These have in turn caused people to create and become engaged in earlier locally unknown activities such as, for example, mushroom gathering. The forces behind encouraging Swedish and Norwegian mushroom foraging are mostly fungi experts, authorities, and today increasingly cooks and influencers who utilize the internet. The process has been partly induced by national and local authorities, but as seen above, the popularity of funnel chanterelle is mainly a result of the work by mushroom activists and experts since the 1970 s [54, 55]. The Scandinavian interest in macrofungi as food is indeed the opposite of vanishing knowledge: in the case of edible mushrooms, several generations of Scandinavians have gradually increased their knowledge since mushroom consumption was first encouraged in the eighteenth century by naturalists [3, 5–7]. This knowledge includes today, as data from our questionnaire shows, awareness of where to find mushrooms, locate habitats, detect and identify, collect and use them, and from our encounters and exchanges we observe that many mushroom gatherers are ready to share their knowledge with others, thus spreading the information wider. “Once you find them, you soon find a lot”, one informant explained about funnel chanterelle in our survey.

Among the ecosystem services provided by macrofungi, cultural services are nowadays highly important for the urbanized population, in contrast to earlier: except for a small circle of French-influenced nobility, ordinary people were cautious and skeptical until a few decades ago [5, 6]. Public interest in mushrooms have skyrocketed now into becoming an important dimension of the Scandinavian landscape experience, recreation and well-being [31, 42]. Mushrooms provide a variety of cultural services [56], not only as they are being harvested, traded, and used as food, but also for recreational, emotional, and aesthetic experiences, and also other subtle ritual and neo-religious dimensions of “just being” in and enjoying the forest [57, 58]. Our data confirms that the typical Swedish mushroom gatherer collects fungi as an aspect of spending time in nature, and enjoying self-foraged, fine-tasting foods; according to our observations, it is much the same with Norwegian mushroom gatherers. Taken together, these factors are crucial to species integration into modern eating habits and leisure activities. Easy availability and preparation are key elements for popularity today: funnel chanterelles are easy to identify, harvest in large quantities, uncomplicated to prepare and consume, and they fit into the comfortable lifestyle and fast eating habits of modern urban Scandinavians. Gathering mushrooms in the forest is even considered an important part of the culture in Sweden and Norway [6, 7]. There is no risk of over-harvesting mushrooms in the forest; forest ecological analyses show that only a small part of all mushrooms growing in the forest are used by humans [12].

Interest in wild mushrooms for human consumption is growing throughout Europe in the wake of culinary authors and TV and internet presenters introducing wild foods; knowledge of edible species is spread through various channels from books and brochures to the web [59]. Consumers and foragers also outside Scandinavia consume *Craterellus tubaeformis* [60]. In neighboring countries, it is popular in Finland [61], and recently it has become more common as food in Estonia. The species is mentioned in guidebooks and writings among others from Denmark, France, Germany, Great Britain, Spain, Switzerland and the USA [44, 60–63]. Its use as human food is confirmed by ethnomycological research from forest and highland areas in different countries in Eurasia and North America [64]. Still, it remains a relative newcomer everywhere in northern European cuisine [65]. Mycologists in Germany promoted it as early as the 1920 s for flavoring [66], and Scandinavian mycologists drew inspiration from Germany when they began to explore its uses; German and Scandinavian languages are close and the countries share much common history. *C. tubaeformis* was mentioned as an edible species in the state of New York by state botanist Charles Horton Peck around 1900, although it appeared to be of no importance [67]. The dissemination of knowledge between academic tradition and the public is still an unexplored area, especially in terms of the economic and practical uses of biological resources in the past [68, 69].

Norwegian and Swedish cuisines are still partly influenced by their traditional prerequisites, but eating habits have changed drastically from the earlier self-subsisting rural diet. Urbanization and its consequences, including distancing toward nature and natural environments, and changes in lifestyles, food production and supply, began more than a century ago, but in the second half of the twentieth century the speed of globalization of foods and knowledge about food and eating, adaptation to contemporary taste preferences and flavorings, health awareness, fashions, and using new technical equipment have increased manifold. In recent decades, together with growing affluence in Scandinavian societies, these factors contributed to more focus on well-being, recreation, health considerations, interest in clean and unprocessed foods, etc., and fashions including wild and local foods have been designed, which reach even luxury restaurants. Our food choices are dictated by a complex range of cultural, economic and social conditions [70], and last but not least–by fashions and information in media and internet. More groups such as urban youth have recently discovered the joy of edible mushrooms, some from family or friends, and others from social media or the internet.

Wild foods are now an important aspect of modern Scandinavian cuisine; funnel chanterelles have truly become a modern “ethnic” food, fitting and seamlessly integrating into the local tastes and cuisines focusing on potatoes, game, veal, fish and sauces [71]. Gathering funnel chanterelles are now well-integrated as a seasonal recreational activity, and recipes containing the mushroom are common in the cuisine at all culinary levels from home cooking to restaurants [72].

## Conclusions

Following the paths of how *Craterellus tubaeformis* became accepted as an edible and useful fungus among Scandinavian mushroom gatherers, we observe that despite being mentioned in mushroom handbooks already at the beginning of the twentieth century in Sweden and Norway, only after the 1970 s it became popular. It took a long time to be recognized and accepted by the general public, but once reaching the awareness of foragers, it has rocketed into vast popularity. At the beginning of the twentieth century, mushroom promotion was part of public campaigns diversifying newly urbanized inhabitants’ diets. By the end of the century it became an element in the efforts to identify “our own” local foods, at a time when Sweden and Norway learned to eat pizzas and Chinese takeaway food. The popularity of funnel chanterelle is partly due to its versatility and partly for its taste: it can be combined with all sorts of dishes, eaten as a side-dish or used as a seasoning for a wide range of traditional and modern dishes: pork, beef, lamb, game (elk, roe deer, reindeer, grouse), fish, pasta, omelets, pies, hot sandwiches, vegetarian dishes, sauces, etc. It can be fried, added to sauces for pasta, stews, gratins and pies; it is much appreciated in soups, too. After just a few decades of introduction, the urban population today–in contrast to their rural ancestors–perceives mushrooms and various other wild foods as a normal part of the diet.

The interest in the species, as well for other macrofungi, has dipped and again reappeared during the past fifty years, but there is no doubt that mushroom gathering remains a popular recreational activity in Scandinavia. Most people know and pick only a few species, but *C. tubaeformis* is one of the most popular mushrooms and the only one available for a long time in late autumn and early winter; now climate change is prolonging the gathering season. It is an economically valuable, sustainable forest resource available to everyone, including tourists who do not need specialized knowledge as the mushroom is easy to recognize. Once this mushroom and its habitat is learned, a forager can expect a considerable seasonal harvest. The species is also inexpensive compared to other mushroom taxa in market stalls and stores. The possibilities for cooking are extensive and this is certainly a fungus for the future.

This study reflects and confirms that contemporary human-fungi relations in Scandinavia contain multiple meanings: mushrooms are food, but mushroom gathering is a recreational activity, and a way of maintaining emotional and cultural ties to the forests and nature among a highly urbanized population. Our results should be able to serve as basis for further studies, especially concerning other species that are or may be of interest to modern consumers. We highlight the need for further interdisciplinary ethnomycological research to explore the importance of wild mushrooms and their potential in modern cuisine, and the necessity of biodiversity understanding and conservation in a time of rapid climate change. Well-managed forests are essential for the survival of a range of interconnected ecosystems of which wild fungi are one, with much to offer humans both in leisure pursuits and gastronomy.

## Data Availability

The authors confirm that the data supporting the findings of this study are available within the article. The responses to the Swedish 2017 questionnaire are available from the Institute for Language and Folklore, Uppsala, Sweden: DFU 41174.
